# Microbial interactions between climate warming and antimicrobial resistance threaten soil carbon storage and global health

**DOI:** 10.1093/ismejo/wraf220

**Published:** 2025-10-04

**Authors:** Shamik Roy, Marc G Dumont, James A Bradley, Marcela Hernández

**Affiliations:** School of Biological Sciences, University of East Anglia, Norwich NR4 7TJ, United Kingdom; Chair for Forest Zoology, Technische Universität Dresden, Tharandt 01737, Germany; School of Biological Sciences, University of Southampton, Southampton SO17 1BJ, United Kingdom; Aix Marseille Univ, Université de Toulon, CNRS, IRD, MIO, Marseille, France; School of Biological and Behavioural Sciences, Queen Mary University of London, London, United Kingdom; School of Biological Sciences, University of East Anglia, Norwich NR4 7TJ, United Kingdom

**Keywords:** soil carbon, carbon use efficiency, antimicrobial resistance, climate warming, microbial communities, soil biogeochemistry

## Abstract

Anthropogenic activities are impacting the environment in ways that may intersect and have compounding effects. In soil, the spread of antibiotics and resistant microbes, and thereby antimicrobial resistance (AMR), can accelerate because of climate change and anthropogenic activities. Here we propose that the dual production and release of antimicrobial compounds to the environment, and the increase in global temperatures as a consequence of climate change, will have synergistic effects leading to both enhanced climate change and disease risk. We predict that an increase in AMR will reduce microbial carbon use efficiency (CUE) because interactions amongst microbes will lead to the allocation of available resources towards AMR and metabolism instead of growth. This reduction in CUE may lead to increased greenhouse gas release; however, the extent to which AMR can affect the stability of soil carbon by altering microbial CUE remains unknown. This concern is especially pertinent in the Arctic, which is warming faster than anywhere else on Earth and contains substantial soil carbon reservoirs.

## Introduction

Antimicrobial resistance (AMR) is a global health concern and is predicted to cause 300 million deaths and up to $100 trillion loss to the global economy by 2050 [1]. It was estimated that nearly 65 000 t of antibiotics were used in livestock farming in 2010, and this has been projected to rise to 105 000 t by 2030 [2]. In humans, widespread resistance to last-resort antibiotics threatens our ability to treat common infections [3]. Antibiotics applied to humans and livestock can, within a few hours, enter the environment either unaltered or as active metabolites that spread AMR [4]. Antibiotics also pose an unmitigated environmental risk because they alter microbial communities in natural environments responsible for driving global biogeochemical cycles [5, 6]; however, the mechanisms by which antibiotics affect microbial carbon (C) and nutrient cycling through AMR remain unknown.

Soil is one of the largest and most diverse habitats on Earth, and it is fundamental to global net primary productivity and food production, amongst other ecosystem services. It also serves as a major reservoir of organic C, storing roughly 2500 Gt – more than three times the C in the atmosphere [7]; however, these functions are increasingly threatened by natural and anthropogenic pressures, including the spread of AMR. Soil microbial communities harbour an extensive reservoir of antimicrobial resistance genes (ARGs), with up to 30% of all known different ARGs represented in soil metagenomes [8, 9]. These genes are widespread across terrestrial biomes and have even been detected in remote regions such as the Arctic [10–12]. Whilst AMR is primarily discussed as a human health crisis, it also has the potential to disrupt the ecological functions of microbial communities that underpin soil carbon cycling.

Climate warming poses an additional, and perhaps more widely recognized threat to long-term soil-C storage. The Arctic, in particular, is undergoing rapid warming, possibly crossing climatic tipping points that could trigger widespread permafrost thaw and microbial decomposition of previously frozen carbon [13]. Rising temperatures can accelerate microbial metabolism [13], enhancing organic matter decomposition and increasing greenhouse gas emissions from soil [14]. In parallel, antibiotic pollution alters microbial composition and interactions, potentially undermining key soil processes such as organic matter stabilization. Despite these converging stressors, the integrative effects of AMR and climate warming on soil microbial communities – and their consequences for soil-C persistence remain largely unexplored.

## Pathways for antibiotic entry into soil

Antibiotic production and resistance amongst microbes are ancient phenomena, having evolved naturally. For example, ARGs, including those effective against β-lactams, tetracyclines, and glycopeptides, were discovered in pristine 30 000-year-old permafrost soil from the Alaska/Yukon region, which predates the societal production and use of antibiotics by tens of thousands of years [15]. Nevertheless, the industrial development of antibiotics for medical intervention has interrupted this natural evolutionary process. As a result, pristine ecosystems with low antibiotic concentrations and naturally occurring AMR are increasingly being subjected to enhanced antibiotic concentrations and accelerated AMR spread [16]. Antibiotics have contributed to a rise of AMR in the environment, which now constitutes a global public health crisis.

The pathways by which industrial antibiotics enter soil are numerous and vary in intensity across ecosystems. In agricultural ecosystems, manure from livestock treated with antibiotics to enhance yield is often used as fertilizer [17]. The manure can contain unmetabolized antibiotic residues and AMR microbes. In grazing ecosystems, which cover ~40% of the Earth’s ice-free land, animal excreta can directly enter the soil, creating localized areas of high antibiotic concentration [18]. These hotspots exert selective pressure on soil microbiota, promoting the proliferation of resistant strains. Other pathways for antibiotic entry into soils include irrigation with treated or untreated wastewater [19], improper disposal of unused or expired antibiotics – either in landfills or through sewage systems [20], and the deposition of airborne particles containing anthropogenically derived antibiotics (from antibiotic volatilization from manure or wastewater, or emissions from pharmaceutical manufacturing) onto soil surfaces through wet (rainfall) or dry deposition, leading to diffuse contamination over broad and even remote areas [21]. Wild birds and animals can also carry and disseminate AMR (through zoonotic spread) from human-dominated landscapes to more pristine ecosystems [22, 23].

The delivery of antibiotics to soil can be sufficient to develop and spread AMR, even at sub-inhibitory concentrations of antibiotics (as low as ng/kg to μg/kg) [24]. When antibiotics enter environments, the lowest concentration where resistance is favoured – known as the minimal selective concentrations (MSCs) – can be orders of magnitude lower than minimal inhibitory concentrations (MICs) [17, 25]. Therefore, even at ultra-low concentrations, antibiotics can affect non-target microbial populations, disrupting ecosystem functions [26]. Persistent antibiotics – compounds that resist degradation in soil and water due to chemical stability, low biodegradability, or strong sorption to particles – can remain bioavailable for weeks to months, continuously exerting selective pressure for AMR [27].

**Figure 1:**
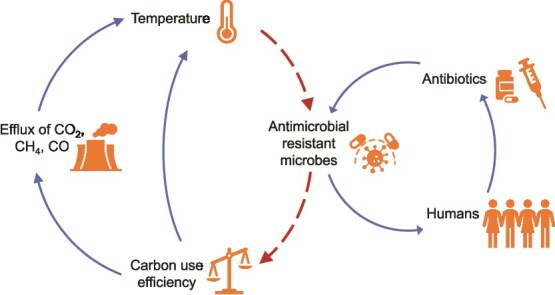
A perspective of the climate feedback loop of temperature, AMR, and microbial carbon use efficiency (dotted lines are hypothesized pathways).

In this perspective, we discuss the interconnected effects of warming and AMR on soil-C dynamics and ecosystem function. We hypothesize that the interplay between climate warming and AMR may create a feedback loop where microbial community shifts not only contribute to increased greenhouse gas emissions but also enhance the spread of AMR (Fig. 1). In the following sections, we explore the mechanisms linking these challenges. First, we examine how warming influences soil-C dynamics, focusing on microbial CUE and its role in C storage versus respiration. Second, we investigate the effect of warming on the spread of AMR, particularly through increased microbial turnover and horizontal gene transfer. Third, we explore how AMR may have repercussions on C cycling, altering microbial resource allocation, diversity, and function in ways that reduce CUE and destabilize C pools. We then discuss the compounding effects of these interactions, including potential ecosystem-dependent variations and seasonal dynamics. Finally, we discuss potential management approaches to safeguard soil health in a warming world.

## Warming and carbon

Soil-C storage and sequestration in response to climate warming is well-studied, and there is a consensus that soil transitions from a C sink to a source even when subjected to a moderate rise in temperature [28, 29]. The global loss of topsoil C under 1°C warming by 2050 is projected to be 30–203 Pg C [28]. The flux of C between soil and atmosphere is sensitive to temperature due to changes to both the C inputs to the soil (through plant production and litter) and outputs from the soil (through microbial degradation of organic C). Warming can increase C input to soil through enhanced plant productivity, a longer growing season, and increased photosynthesis rates [30]. In contrast, rates of microbial decomposition of soil organic matter are also enhanced under warming [31], potentially offsetting increased C inputs. A key mechanism underlying this effect is the direct influence of temperature on microbial extracellular activity: extracellular enzymes produced by microbes become more sensitive with increased temperature, accelerating the breakdown of organic matter and stimulating respiration [32]. This temperature sensitivity arises due to 1) shifts in microbial abundance and community composition [33]; 2) priming effects that enhance microbial decomposition through increased root exudation and labile C inputs [34] and 3) microbial acclimation (or lack thereof) to warming, which alters microbial CUE and the partitioning of C between biomass production and respiration [35]. Microbial CUE – a central determinant of soil organic C (SOC) storage – represents the fraction of assimilated C that is allocated to new microbial biomass [36]. Microorganisms with a high CUE allocate a greater proportion of their C uptake into biomass, promoting SOC accumulation. In contrast, low CUE diverts more C to respiration, leading to losses and increased CO_2_ emissions [37]. Warming also accelerates the decomposition of older, more stable C pools, further reducing soil-C storage [31]. Physical changes to C accessibility, such as the thawing of permafrost, will also make centuries-old frozen soil C vulnerable to microbial decomposition [38, 39]. The magnitude of C release from soils to the atmosphere depends on regional factors such as moisture, ecosystem type, and rates of warming [40, 41]. Localized studies are crucial to assessing the effect of prolonged temperature rise on soil C. Meanwhile, robust upscaling methods are needed to overcome large uncertainties introduced in making global assessments and climate model simulations [42], which also hinder the framing and implementation of climate stewardship policies.

## Warming and AMR

Prolonged rises in global temperatures can increase AMR by increasing the relative abundance of microbes that naturally harbour antibiotic-resistance genes [43–45], as well as increasing the rate of horizontal gene transfer (HGT) [46] – thereby increasing the number of antibiotic-resistant organisms as well as the number of antibiotics against which these organisms develop resistance. Warming can also induce metabolic and physiological changes in microbes at the cellular level, which may co-activate pathways for antibiotic tolerance. These changes could include alterations in the cell membrane composition, protein-folding machinery, and the upregulation of stress responses [47, 48]. Whilst these pathways can provide broad-spectrum tolerance, they are distinct from genetic AMR, which involves horizontally acquired or mutational traits that confer heritable drug resistance. The co-activation of shared stress pathways could, in some cases, increase microbial efficiency by streamlining cellular responses to multiple stressors; however, many of these responses – particularly efflux systems and protein quality control machinery – incur substantial energetic costs when chronically activated. As a result, under sustained warming and antibiotic exposure, the energetic demands of maintaining resistance traits and activating general stress responses may reduce microbial CUE, even when some molecular pathways are shared. Additionally, recent studies have shown that warming-induced increases in organic matter decomposition can release heavy metals, which are otherwise protected in the soil [49]. These heavy metals can lead to increased metal resistance, which may act as selective agents for the spread of AMR [50]. Climate warming also increases the prevalence of pathogenic bacteria and disproportionately affects developing countries [51–53]. In response, nations – especially those facing higher disease burdens – may increasingly rely on antibiotics to manage infections exacerbated by climate-related changes, potentially accelerating AMR.

## AMR and carbon

We predict that increasing AMR will reduce microbial CUE, deplete SOC stocks, and enhanced CO_2_ production from soils. Under selective pressure from AMR, microbial communities may allocate a disproportionate share of cellular resources to resistance, maintenance, and survival, rather than growth. These energetic costs include the synthesis and regulation of efflux pumps, enzymes that degrade antibiotics, and other protective proteins. Additionally, HGT of resistance genes (via plasmids or transposons) imposes additional metabolic demands on recipient cells [12], resulting in reduced CUE with ecosystem-level consequences for soil C. A recent meta-analysis estimated that antibiotics reduced microbial biomass by 17%, with the magnitude of reduction varying by the latitude from which the soil was collected [54]. Another meta-analysis reported that the fitness cost of AMR ranges from 1% to 50%, depending on the mode of acquisition [55]. This cost reflects the difference in microbial growth with and without antibiotic exposure.

As microbial CUE declines, less C is incorporated into biomass or stabilized as recalcitrant organic matter through microbial necromass, whilst more is respired as CO_2_. Although necromass is not inherently stable, it can persist through interactions with soil minerals, incorporation into aggregates, or protection within physically inaccessible microsites. Consequently, over time, reduced microbial CUE may lead to lower soil-C sequestration, as a greater proportion of carbon is respired rather than incorporated into microbial biomass or stabilized organic matter [56]. Experimental evidence supports this hypothesis: microbial communities exposed to antibiotics, often with elevated resistance gene levels, have been shown to respire more CO_2_ per unit biomass than unexposed controls, indicating a lower CUE [6, 57]; however, the influence of AMR on soil-C dynamics is likely to be complex and context-dependent, varying across ecosystems and environmental conditions.

Soil C may also influence AMR through feedbacks between both the quality and quantity of soil C, and the structure and function of microbial communities (Box 1). For example, high-quality or easily degradable C sources can stimulate microbial growth and activity, thereby increasing opportunities for HGT of ARGs [12]. In contrast, low-quality or recalcitrant C tends to select for microbial taxa that are adapted to resource-limited conditions, which may in turn shape the resistance profiles of these communities [58]. Variations in soil-C content can also modulate microbial interactions, such as competition and cooperation, that influence the persistence and spread of resistance traits [59]. As such, the concept of “healthy soils”, particularly through the sustainable management of soil C, represents a promising pathway for AMR mitigation and stewardship [60].

### Box 1. Case study and emerging questions

In a recent field experiment, Lucas *et al.* [61] examined the combined effects of elevated temperature and antibiotic exposure on microbial contributions to soil-C cycling. The study revealed that these stressors jointly reduced bacterial network complexity and promoted fungal dominance, likely due to decreased competition and increased nutrient availability from bacterial necromass. This shift led to a lower microbial C:N ratio, with potential long-term implications for soil-C stabilization. This example illustrates how intersecting stressors can restructure microbial communities in ways that affect CUE and SOC stabilization. Although the study did not measure AMR directly, antibiotic exposure likely selected for resistant taxa, raising broader concerns about long-term community metabolic trade-offs within affected communities; however, the broader applicability of these findings remain uncertain, as natural soils are simultaneously exposed to multiple stressors, including fungicides, heavy metals, and land-use change. Key open questions include: To what extent can environmental stressor–induced restructuring of microbial communities mitigate the negative effects of those stressors on the ecosystem? Could legacy effects or ecological memory within AMR-affected communities attenuate immediate respiratory responses to warming? Finally, how generalizable are these patterns across diverse ecosystems and longer temporal scales?

## Compounding effects of AMR, warming, and carbon

Both AMR and climate warming independently shape soil microbial communities in ways that influence soil C cycling. Prior studies have shown that both elevated temperatures and antibiotic exposure can reduce microbial CUE [5, 62], and both stressors are known to alter AMR dynamics; however, the nature of their combined impact on soil microbial processes remains unresolved. We hypothesize that warming-induced microbial shifts, compounded by increased antibiotic exposure, may promote resource allocation towards resistance and maintenance rather than growth. This reallocation could reduce microbial CUE and enhance soil C loss. If realized, such changes could accelerate both global warming and the spread of AMR – posing compounding challenges for environmental and public health. The effects of warming and AMR on microbial CUE and soil-C loss may be non-redundant and potentially synergistic. In an additive scenario, the combined effect on microbial communities or soil C equals the sum of the individual impacts. In a multiplicative scenario, the joint effect is the product of their individual effects. When the observed impact is significantly greater or less than expected under either of these models, it is considered an interaction – which may be synergistic (greater than expected) or antagonistic (less than expected). In the context of soil C, a synergistic interaction would imply that warming and AMR together reduce CUE and accelerate C loss more than predicted based on their independent impacts. This is particularly concerning for systems already vulnerable to warming, such as permafrost regions, or those heavily impacted by antibiotics, such as agricultural soils. Understanding whether warming and AMR interact additively, multiplicatively, or synergistically is critical for accurately predicting soil-C feedbacks under future climate and antibiotic-use scenarios. In the following section, we examine how these interactions may vary across ecosystems.

## Ecosystem-dependent effects

The impacts of AMR, C cycling, and climate warming are unlikely to be uniform across ecosystems. We highlight several ecologically significant regions – such as the Arctic, tropical grasslands and forests, and agricultural systems – where the interactions amongst these factors may be especially pronounced.

(i) Arctic soils

Arctic permafrost soils store an estimated 1672 billion tons of C – more than twice the amount currently present in the atmosphere [63]. This vast C reservoir is highly vulnerable to microbially mediated degradation, a process that accelerates under warming and results in the release of CO_2_ and CH_4_ [64–66]. Arctic warming is intensifying due to polar amplification, occurring at three to four times the global average [67, 68]. Even the release of just 1% of this stored C could increase atmospheric CO_2_ concentrations by 6.6–8.7 ppm [63] – three to four times the current annual rise – pushing CO₂ levels further into the 425–785 ppm range associated with a 1.5°C warming threshold [69].

The Arctic is often considered pristine compared to more populated regions but the detection of both ancient and anthropogenic ARGs suggests a more complex baseline. Studies have documented ARGs – even clinically significant ones – in remote Arctic environments, highlighting pathways of human-linked dissemination (e.g. via migratory wildlife and wastewater spread) [12, 70–72]. Accelerating environmental and human pressures are likely to enhance the emergence and spread baseline of AMR in the Arctic. Thawing permafrost, expanding human activity (e.g. shipping, infrastructure, tourism), limited and costly treatment of wastewater in cold and remote regions, and increasing connectivity through waterways and migratory species all contribute to the risk of antibiotic dissemination in the region [12, 71, 72]. Even sub-inhibitory concentrations of antibiotics can select for resistance traits and disrupt microbial networks [15]. These changes may restructure microbial communities – favouring resistance maintenance over growth – which could reduce microbial CUE and enhance soil carbon loss; however, the extent to which AMR will spread in the Arctic, and how it may interact with climate warming and soil C dynamics, remains largely unknown.

(ii) Tropical ecosystems

Tropical grasslands and forests are undergoing extensive and rapid land-use change to support growing human populations. Tropical grasslands alone store ~10%–30% of global soil C [73], whilst trees in tropical forests sequester an estimated 200–300 Pg C, roughly a third of the C currently held in the atmosphere [74]. C fluxes in these regions exhibit high interannual variability, primarily driven by fluctuations in temperature and precipitation. As these ecosystems undergo land-use change to pasture lands or urbanization, AMR can also increase, contributing to variable carbon exchanges.

Tropical countries report some of the highest rates of antibiotic use globally [75]. These overlapping stressors – climate variability, land-use change, and antibiotic pressure – may jointly intensify microbial activity and reduce microbial CUE, potentially amplifying microbially driven soil-C loss [76]; however, the magnitude and direction of this impact remain highly uncertain. We hypothesize that the mechanisms by which AMR and warming influence soil-C dynamics in tropical systems are highly localized and dependent on ecosystem context, microbial composition, and land-use history.

(iii) Agricultural lands

Agricultural systems constitute the largest human-managed land use globally [77], and are increasingly shaped by the application of organic fertilizers such as livestock manure and sewage sludge. Whilst these inputs are often promoted as sustainable, they are also major vectors for ARGs [76]. The widespread use of antibiotics at sub-therapeutic doses in livestock production to enhance livestock growth and health contributes substantially to the introduction of AMR into soil [76]. Even in C-depleted agricultural soils, resistance traits can proliferate due to high microbial turnover, resource inputs, and selective pressure from sub-therapeutic antibiotic residues. These lands are also vulnerable to warming-induced C losses that feed back to AMR–carbon–warming interactions. Substantial inputs of manure and fertilizers can enhance soil-C storage by increasing microbial CUE [78, 79], but the spread of AMR in agricultural soils may reduce microbial CUE and thereby limit these benefits. Moreover, manure addition can also have a negative effect on soil-C [78], as the large quantity of labile C from manure primes already C-depleted soils for further C loss to the atmosphere – creating a feedback loop that accelerates both warming and the spread of AMR. This dynamic threatens both the ecological sustainability and economic viability of agriculture, as C lost through AMR and warming must be replenished using costly organic fertilizers. We propose that increased resistance within microbial communities – driven by antibiotic exposure and elevated temperatures – may reduce microbial CUE and accelerate soil-C depletion in agricultural landscapes. This intersection of environmental and anthropogenic pressures underscores the urgent need for integrated management strategies that simultaneously promote soil health and mitigate resistance spread.

(iv) Priorities

The ecosystem-specific interactions between AMR, warming, and soil C underscore the complexity of this emerging research frontier. Despite growing recognition of this feedback, significant knowledge gaps remain. We outline key research priorities in Box 2 to guide future empirical and modelling efforts.

### Box 2. Research priorities at the AMR–warming–carbon nexus

1. Mechanistic insight: Critical gaps remain in the mechanistic understanding of the combined AMR and warming-related mechanisms impacting microbes and soil C. Some key questions include: a) How do microbial metabolisms differ between soil microbial communities exposed to AMR and warming, and those that have not? b) What changes occur to transcriptomic profiles of microbial communities exposed to warming and AMR, if any? c) Do aboveground plants respond differently in plots exposed to warming and AMR than those that have not been? d) How do microbial communities exhibit resistance and resilience to changes in the ecosystem caused by the joint effects of warming and AMR?
2. Temporal scales: Long-term studies are needed to determine whether combined warming and AMR lead to sustained soil-C loss or microbial adaptation and stabilization.
3. Spatial scales: Most soil manipulation experiments are limited to small plots (~10^1^ m^2^); scaling to landscape levels (10^4^–10^6^ m^2^) is essential for broader-scale applicability and ecosystem-level relevance.
4. Context specificity: Can mechanisms observed at the level of one ecosystem be generalized to other ecosystems? Do different antibiotics, or combinations of antibiotics, interact with warming and affect ecosystem dynamics differently? Studies are needed across diverse biomes and ecoregions, testing a range of ecosystem types, antibiotic treatments, and warming scenarios.
5. Carbon, nutrients, and climate-warming gases. In addition to CO_2_, trace gases such as methane (CH_4_) and carbon monoxide (CO) are important components of the C cycle yet are less studied in the context of warming and AMR. These atmospheric gases serve as energy sources to microbial colonizers, thus playing an important role in pedogenesis in pioneer ecosystems [80–82]. Moreover, biogeochemical cycles do not operate in isolation, and thus future studies must consider other elemental cycles, including N and P cycling, in relation to warming-AMR interactions.
6. Data-model integration: To predict the impacts of AMR and warming on soil carbon at scale, data collected across diverse spatial and temporal contexts must be integrated using robust statistical and computational frameworks. Current climate models often struggle to resolve microbial processes and typically do not incorporate emerging stressors such as AMR; however, as AMR increasingly influences microbial community structure and function, particularly under warming, its omission could limit the accuracy of future soil-C projections. Interactions between multiple stressors (e.g. warming and AMR) introduce nonlinearities that are difficult to capture without targeted empirical data and model parameterization. We recommend that climate and Earth system modellers begin to incorporate AMR-related microbial processes, in close collaboration with empirical scientists, to improve the predictive power of soil carbon and climate feedback models.
7. Multidisciplinary resolve: Addressing this complex challenge will require joint efforts from ecologists, social scientists, economists, and policymakers. Promising directions include:
  a) reducing antibiotic inputs,
  b) soil amendments to reduce bioavailable antibiotics,
  c) local land-use strategies to buffer warming,
  d) coordinated AMR monitoring, and
  e) global initiatives (e.g. “One Health”, 4 per 1000) to align mitigation goals.

## Conclusion and future directions

We propose that an integrated, interdisciplinary framework – combining environmental monitoring, microbial functional profiling, and long-term field experiments – is essential to disentangle the interactions between AMR, climate warming, and soil-C cycling. We argue that this intersection represents a critical and understudied research frontier in microbial ecology.

Understanding how microorganisms mediate the coupled dynamics of AMR, climate warming, and soil-C fate is vital for accurately forecasting soil-C trajectories and developing strategies to mitigate both climate change and AMR risks. We emphasize that AMR should not be viewed solely as a public health crisis, but also as an ecological disruptor with global biogeochemical consequences.

Future research and policy must address warming and AMR in tandem to develop effective, scalable solutions that preserve soil function, safeguard C stocks, and ensure ecosystem resilience under a changing climate.

## Data Availability

No datasets were generated in this work.
